# Low-Input RNA-Sequencing in Patients with Cartilage Lesions, Osteoarthritis, and Healthy Cartilage

**DOI:** 10.1177/19476035211057245

**Published:** 2021-11-15

**Authors:** Katherine Wang, Q.Y. Esbensen, T.A. Karlsen, C.N. Eftang, C. Owesen, A. Aroen, R.B. Jakobsen

**Affiliations:** 1 Faculty of Medicine, University of Oslo, Oslo, Norway; 2 Oslo Sports Trauma Research Center, Norwegian School of Sports Sciences, Oslo, Norway; 3 Department of Clinical Molecular Biology (EpiGen), Akershus University Hospital, Lørenskog, Norway; 4 Department of Clinical Molecular Biology, University of Oslo, Oslo, Norway; 5 Norwegian Center for Stem Cell Research, Department of Immunology and Transfusion Medicine, Oslo University Hospital, Rikshospitalet, Oslo, Norway; 6 Department of Pathology, Akershus University Hospital, Lørenskog, Norway; 7 Department of Orthopaedic Surgery, Akershus University Hospital, Lørenskog, Norway; 8 Institute of Clinical Medicine, Faculty of Medicine, University of Oslo, Oslo, Norway; 9 Department of Health Management and Health Economics, Institute of Health and Society, Faculty of Medicine, University of Oslo, Oslo, Norway

**Keywords:** knee cartilage, healthy cartilage, cartilage lesion, osteoarthritis, gene expression

## Abstract

**Objective:**

To analyze and compare cartilage samples from 3 groups of patients utilizing low-input RNA-sequencing.

**Design:**

Cartilage biopsies were collected from patients in 3 groups (*n* = 48): Cartilage lesion (CL) patients had at least ICRS grade 2, osteoarthritis (OA) samples were taken from patients undergoing knee replacement, and healthy cartilage (HC) was taken from ACL-reconstruction patients without CLs. RNA was isolated using an optimized protocol. RNA samples were assessed for quality and sequenced with a low-input SmartSeq2 protocol.

**Results:**

RNA isolation yielded 48 samples with sufficient quality for sequencing. After quality control, 13 samples in the OA group, 9 in the HC group, and 9 in the CL group were included in the analysis. There was a high degree of co-clustering between the HC and CL groups with only 6 genes significantly up- or downregulated. OA and the combined HC/CL group clustered significantly separate from each other, yielding 659 significantly upregulated and 1,369 downregulated genes. GO-term analysis revealed that genes matched to cartilage and connective tissue development terms.

**Conclusion:**

The gene expression profiles from the 3 groups suggest that there are no major differences in gene expression between cartilage from knees with a cartilage injury and knees without an apparent cartilage injury. OA cartilage, as expected, showed markedly different gene expression from the other 2 groups. The gene expression profiles resulting from this low-input RNA-sequencing study offer opportunities to discover new pathways not previously recognized that may be explored in future studies.

## Introduction

Cartilage damage and degeneration are some of the most common health-related issues affecting today’s aging population.^1^ Isolated cartilage and osteochondral lesions of the knee present a difficult clinical challenge, especially in younger patients for whom alternatives such as partial or total knee replacement (TKR) are rarely advised. Age, obesity, biomechanical instability, and genetics are part of the multifactorial etiology of osteoarthritis (OA).^2^ It is also well established that injury to the knee joint leads to earlier development of OA than in those without previous injury.^3–5^ However, little is known about this process that leads to the earlier development of OA.^2^ Spontaneous healing is seen infrequently after traumatic cartilage injury. The tissue filling the defect generally comprises fibrocartilaginous tissue instead of normal hyaline cartilage.^6^

Multiple treatment modalities have been developed for cartilage lesions (CLs) spanning from refixation of chondral fragments to stem cell treatment.^7^ Some treatments, such as autologous chondrocyte implantation (ACI), have been promising in the short and middle term, but often also result in the patient developing fibrous cartilage instead of native hyaline cartilage.^8^ Tissue engineering using stem cells has been proposed as a promising future solution for both OA and traumatic CLs. However, current results demonstrate many of the same issues as implantation of chondrocytes, with cells differentiating into a hypertrophic chondrocyte profile generating fibrous cartilage.^9^

In the last few decades, studies have identified multiple genes associated with OA disease progression,^1,10,11^ but treatment options for OA remain limited apart from TKR. Three main OA-associated pathways have been identified: extracellular matrix degradation, collagen catabolism, and angiogenesis,^10^ where the presence of pro-inflammatory cytokines is postulated to inhibit matrix synthesis and promote increased production of matrix degrading enzymes.^12^ With RNA-sequencing, studies have shown a large number of differentially expressed genes when comparing normal cartilage from cadaver donors with OA samples.^13,14^ Similarly, Coutinho de Almeida *et al.*^15^ found over 140 miRNAs and 2,300 mRNAs to be differentially expressed between lesioned and preserved OA articular cartilage in the same knee. However, Lewallen *et al.*^16^ reported that OA development is concomitant with whole-joint changes that trigger molecular responses in the undamaged cartilage within the joint. This demonstrates that sampling anatomically intact cartilage from OA joints is not representative of healthy chondrocytes,^16^ and studies comparing OA samples with healthy cartilage (HC) from non-OA knees are rare and living donors even more rare. As a parallel, a biological distinction has been found between traumatic and degenerative meniscus tears with samples from these 2 processes expressing different genes.^17^ There are, however, few studies examining differences between cartilage from a knee with an isolated traumatic lesion as compared to cartilage from knees with only ligamentous injury or to osteoarthritic cartilage.^18^ It is possible that focal CLs could be the first sign of a more generalized disease process in the cartilage, raising the issue of whether cartilage repair is possible. Such information can help to better guide the development of treatments and future clinical decisions.^17^

In this study, we aimed to firmly establish differences and similarities in gene expression based on low-input RNA-sequencing from samples of HC, cartilage from patients with a CL, and cartilage from patients with osteoarthritic cartilage, to provide the basis for future studies. We hypothesized that there would be distinct differences between the gene expression profiles of healthy and OA cartilage, with samples from CLs showing a separate profile from the other 2 groups.

## Methods

### Patient Selection

Knee cartilage samples were obtained from 48 patients, aged between 18 and 65 years, in 3 patient groups; osteoarthritic (OA) samples from patients undergoing TKR, CL samples from patients undergoing arthroscopic knee surgery for cartilage damage with an ICRS score of at least grade 2, and HC from anterior cruciate ligament (ACL) reconstruction patients with cartilage that appeared to have no lesions or signs of degeneration during arthroscopy. There were 16 patients in each group.

### Sample Collection

All samples were taken from the same location within the knee, about 1 cm from the posterior cruciate ligament attachment site on the medial condyle of the femur. From the OA patients, the medial condyle piece was rinsed and cleaned of gross contamination with blood and placed directly on dry ice and the cartilage sample was cut out with a clean scalpel. The HC and CL samples were obtained using a curette by the operating surgeon. We aimed to only remove cartilage without subchondral bone for each sample and were limited to approximately 15 mm^3^ in size to minimize potential risks to patients. All samples were immediately frozen on dry ice and subsequently transported from the operating theater to the laboratory for storage in a −80°C freezer within 5 minutes. There were no treatments used on the samples prior to storage.

### RNA Isolation

mRNA was isolated from each frozen cartilage sample using a combination of a modified TriZol (Thermo Fisher Scientific, Waltham, MA) protocol for lysing samples and separating phases, and Direct-zol RNA Miniprep kit (ZYMO Research, Orange County, CA) for isolation.

Each sample was pulverized using a hammer on dry ice, then further homogenized in 1.3 ml TriZol in a FastPrep-24 (MP Biomedicals, Santa Ana, CA) mixer for 2 rounds of 15 seconds. After snap spin down, 1 ml supernatant was extracted and mixed with 200 μl chloroform and then centrifuged for 15 minutes at 4°C to separate phases. The aqueous layer (550 μl) was mixed with 1,650 μl TriZol and 2,200 μl 100% ethanol to be run through the Zymo-Spin Columns following the Direct-zol protocol and washed accordingly. The column was further centrifuged with a closed cap for 5 minutes to dry the membrane, left to stand for 3 minutes at room temperature, and then eluted with 30 μl RNase-free water. All samples were quality tested using NanoDrop Spectrophotometer ND-1000 for purity, and Bioanalyzer 2100 (Agilent) for integrity. We aimed for an RNA integrity number (RIN) over 5 for each sample (**Table 1**).^19^

**Table 1.** Characteristics of Patients Included in the Study with Comparison of Means for Sex, Age, and BMI between Study Groups.

	HC (16)	CL (16)	OA (16)	All (48)	*P* Value
M/F (% female)	6/10 (63)	12/4 (25)	11/5 (31)	29/19 (40)	0.068
Age (range)	19-48	18-57	53-64	18-64	
Age (mean [SD])	31 (9.7)	36 (11.5)	60 (3.4)	42 (15.5)	<0.001
BMI (mean [SD])	27.4 (4.3)	26.3 (3.0)	31.6 (4.9)	28.4 (4.7)	0.002
ASA 1 (no.)	13	13	1	27	
ASA 2 (no.)	3	3	12	18	
ASA 3 (no.)	0	0	3	3	
RIN (mean)	6.11	5.59	6.27	5.99	

HC = healthy cartilage; CL = cartilage lesion; OA = osteoarthritis; BMI = body mass index;

ASA = American Society of Anesthesiologists classification; RIN = RNA integrity number.

### RNA Sequencing

Smart-Seq2 version 1.1 (Illumina, San Diego, CA) libraries were prepared by Broad Technology Labs and sequenced by Broad Genomics Platform according to the Smart-Seq2 protocol with minor modifications.^20,21^ This method is based on the capture of polyadenylated transcripts and increases both yield and length of cDNA inserts generated from low amounts of input RNA by utilizing full-length transcriptional profiling through reverse transcription, template switching, and pre-amplification. Sequencing (NextSeq500, Illumina) was carried out using High Output kit to generate 2 × 25 bp reads. The data were aligned using STAR v2.4.2a^22^ and processed using Picard v1.1073 (http://broadinstitute.github.io/picard). Genes were then quantified using RSEM v1.2.21^23^ with the paired-end option and the data were quality-assessed using RNA-SeQC.^24^

### Bioinformatic Analysis

The BAM files were uploaded, pre-processed, and analyzed using the SeqMonk software (https://www.bioinformatics.babraham.ac.uk/). Samples were quality controlled for percent in gene (>75%). The DEseq2 package in SeqMonk was used for differential expression analysis and TSNE for clustering analysis. Cut-off values were set more than 2-fold difference in expression values with a false discovery rate (FDR) of less than 5% and an FPKM of at least 1 in one of the groups. For GO-term analysis, the ShinyGO v.0.61 software was used.^25^ For network analysis we used STRING v.11.0 software. Difference in age, sex, and body mass index (BMI) between groups was analyzed using analysis of variance (ANOVA) in IBM SPSS version 25.

### SiRNA and Protein Expression Experiments

#### Isolation and culture of chondrocytes

Articular cartilage was obtained from discarded tissue of patients undergoing knee replacement surgery. Cartilage pieces were taken from a part of the surface of the femoral condyle without macroscopic signs of OA. The cartilage tissue was cut, digested, and cultured as previously described^8^; siRNA experiments were performed on passage 3.

#### siRNA

The Amaxa Nucleofector System and the Amaxa Human Chondrocyte Nucleofector Kit were used for electroporation of siRNAs following the protocols from the manufacturer (Lonza, Walkersville, MD). SiRNA was purchased from Thermo Fisher Scientific: MSMP ID# s57860 and s57861, STC1 ID #12722 and 12815, and control ID# AM4611. Briefly, each reaction consisted of 1.0 × 10^6^ cells, 1 µM of siRNA in a total volume of 100 µl. The cells were seeded in 10% hPLP without antibiotics. After 24 hours the medium was changed to 10% hPLP with 1% penicillin/streptomycin. Cells were harvested for real-time RT-qPCR and Western blot after 48 hours.

#### Isolation of RNA, cDNA synthesis, and real-time RT-qPCR

Total RNA was isolated using the miRNeasy mini kit according to protocols from the manufacturer (Qiagen, Germantown, MD). cDNA synthesis and real-time RT-qPCR were performed following protocols from the manufacturer using the Taqman High-Capacity cDNA Reverse Transcription Kit and Taqman gene expression assays (Thermo Fisher Scientific). Briefly, 200 ng RNA in a total volume of 15 µl was reverse transcribed into cDNA. Technical triplicates were used for real-time RT-qPCR and each replicate contained 0.2 µl cDNA in a total volume of 15 µl. The thermocycling parameters were 95°C for 10 minutes followed by 40 cycles of 95°C for 15 seconds and 60°C for 1 minute. The following probes (Thermo Fisher Scientific) were used: MSMP Hs04195328_g1, STC1 Hs00174970_m1, *GAPDH* Hs99999905_m1, COL1A1: Hs00164004_m1, MMP13 Hs00233992_m1.

#### Western blotting

Cell lysates corresponding to 200,000 cells were loaded onto a 4%-20% gradient or 10% polyacrylamide gel (Bio-Rad, Hercules, CA). Proteins were separated by gel electrophoresis, transferred to PVDF membranes, and incubated with appropriate antibodies (Abcam, Cambridge: SOX9 #Ab76997, PCNA Ab18197, and ACTB Ab8226) before visualizing the bands using the myECL imager (Thermo Fisher Scientific).

### Ethics

All patients gave written informed consent. The study was approved by the Regional Committee for Medical Research Ethics, South-Eastern Norway, Section D. All methods and experiments were performed in accordance with the relevant guidelines and regulations.

## Results

Over a period of 12 months, 48 samples were collected across the 3 patient groups during surgical procedures. The characteristics of the patients are described in **Table 1**. The average age at the time of operation for all patients was 42 (SD 16) years, with the HC group having the lowest mean age at 31 (SD 10) years and the OA group with the highest at 60 (SD 3) years. There was also variation in BMI between the groups from the CL group averaging 27.4 (SD 4.3) kg/m^2^ to the OA group averaging 31.6 (SD 4.9) kg/m^2^. There was a significant difference in the mean age (*P* < 0.001) and BMI (*P* = 0.002) between groups. The American Society of Anesthesiologists (ASA) classification was also used to give a general description of the patients’ health at the time of operation. Patients in the HC and CL group were mostly ASA 1 patients (*n* = 13 each) with a few classified as ASA 2 (*n* = 3 each), while the OA group had mostly patients classified as ASA 2 (*n* = 12).

**Figure 1.**
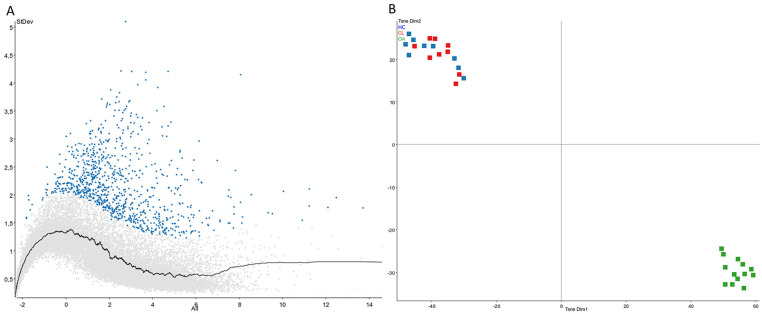
(**A**) Gene variance to identify which genes differ most between samples. Blue genes were selected for clustering analysis (SD >0.7). (**B**) TSNE clustering analysis of genes with greatest variance between samples. Healthy cartilage (blue) and cartilage lesion (red) cluster together and separately from osteoarthritis samples (green). HC = healthy cartilage; CL = cartilage lesion; OA = osteoarthritis.

After quality control, 13 samples remained in the OA group and 9 samples each in the HC and CL groups, giving a total of 31 samples. To investigate heterogeneity, TSNE clustering analysis was performed on the remaining samples (**Fig. 1A and B**). The HC and CL samples clustered closely together, while the OA samples clustered separately. This created 2 separate subpopulations, one with the OA samples and one with the HC and CL samples together. Furthermore, only 6 genes differed significantly between the HC and CL groups (**Table 2**), and we therefore chose to treat these two as one control group in the further analysis.

**Table 2.** Genes with At Least a 2-Fold Change between the Level Expressed in CL Compared with HC Samples.

Gene	Fold Change	FDR	Normalized Count in HC	Link to Cartilage
MSMP	–22.8	0.02	2,706	Encodes a member of the beta-microseminoprotein family, prostate associated. Highly expressed in neonatal articular cartilage and a potential target of miR-140 linked closely to cartilage development.^26^ Has been identified to be downregulated in chondrocytes in response to increased IGF-1.^27,28^
RGP1	–15.5	0.02	304	Encodes for the enzyme UDP-arabinopyranose pyranomutase. Depletion of RGP1 in zebrafish lead to intracellular accumulation of collagen currently investigated in NIH-funded research (https://grantome.com/grant/NIH/F31-DE030007-01).
STC1	–8.1	<0.01	2.1	Encodes a glycoprotein. Has functions in several physiological processes, including bone development, angiogenesis, and modulation of the inflammatory response.^29^
UNC5C	–2.1	0.04	2.3	Belongs to the UNC-5 family of netrin receptors, secreted proteins known to direct axon development and cell migration during neural development.^30^ No known link to cartilage.
ACOT7	2.8	<0.01	1.2	Acyl-CoA thioesterase 7, cytosolic enzyme involved in lipolysis.^31^ No known link to cartilage.
ANGPTL7	3.6	0.04	6.4	Angiopoietin-like protein 7, secreted protein with poorly understood biological function. Has been investigated in glaucoma cells and alters expression of several extracellular matrix molecules including collagen types I, IV, and V and fibronectin.^32^

HC is treated as the control. Sorted for highest expression in the control.

CL = Cartilage lesion; HC = healthy cartilage; FDR = false discovery rate.

### Healthy Cartilage versus Cartilage Lesion

Six genes had at least a 2-fold change between the level expressed in CL compared with HC samples (**Table 2**). Most of these genes have not previously been directly associated with cartilage or cartilage damage. The microseminoprotein, prostate-associated (MSMP) gene was downregulated 22-fold in CL samples, and although it has been characterized as prostate-specific, it has previously been shown to be expressed in chondrocytes and may play a role in the early stages of chondrocyte differentiation.^28,33^ Retrograde Golgi Transport Homolog (RGP1), which encodes an enzyme with no clearly defined function in cartilage, was downregulated 15-fold. Stanniocalcin-1 (STC1) was downregulated more than 8-fold in CL albeit from a low expression and is known to encode a glycoprotein. It has been shown to be involved in a wide variety of physiological processes, including bone development, angiogenesis, and modulation of the inflammatory response.^34^

### Osteoarthritis versus Control Group

**Figure 2.**
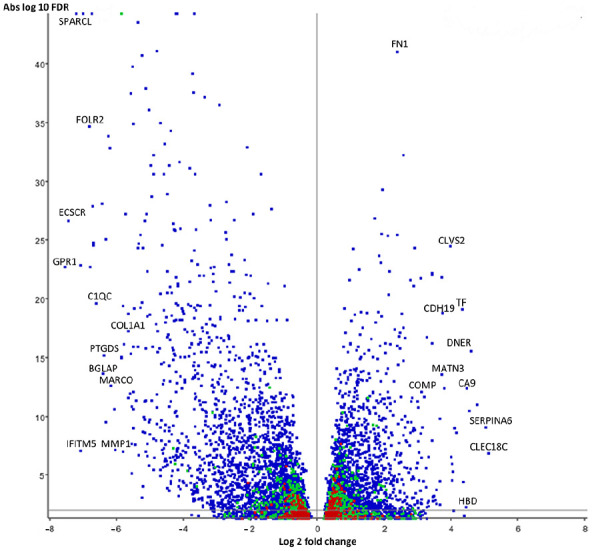
Volcanoplot illustrating up- and downregulated genes in osteoarthritis samples compared to control along the x-axis and FDR along the y-axis. The horizontal line above the x-axis corresponds to FDR = 0.05. Key genes marked. Colors represent overlapping points (density) from highest (red) to lowest (blue). FDR = false discovery rate.

**Figure 3.**
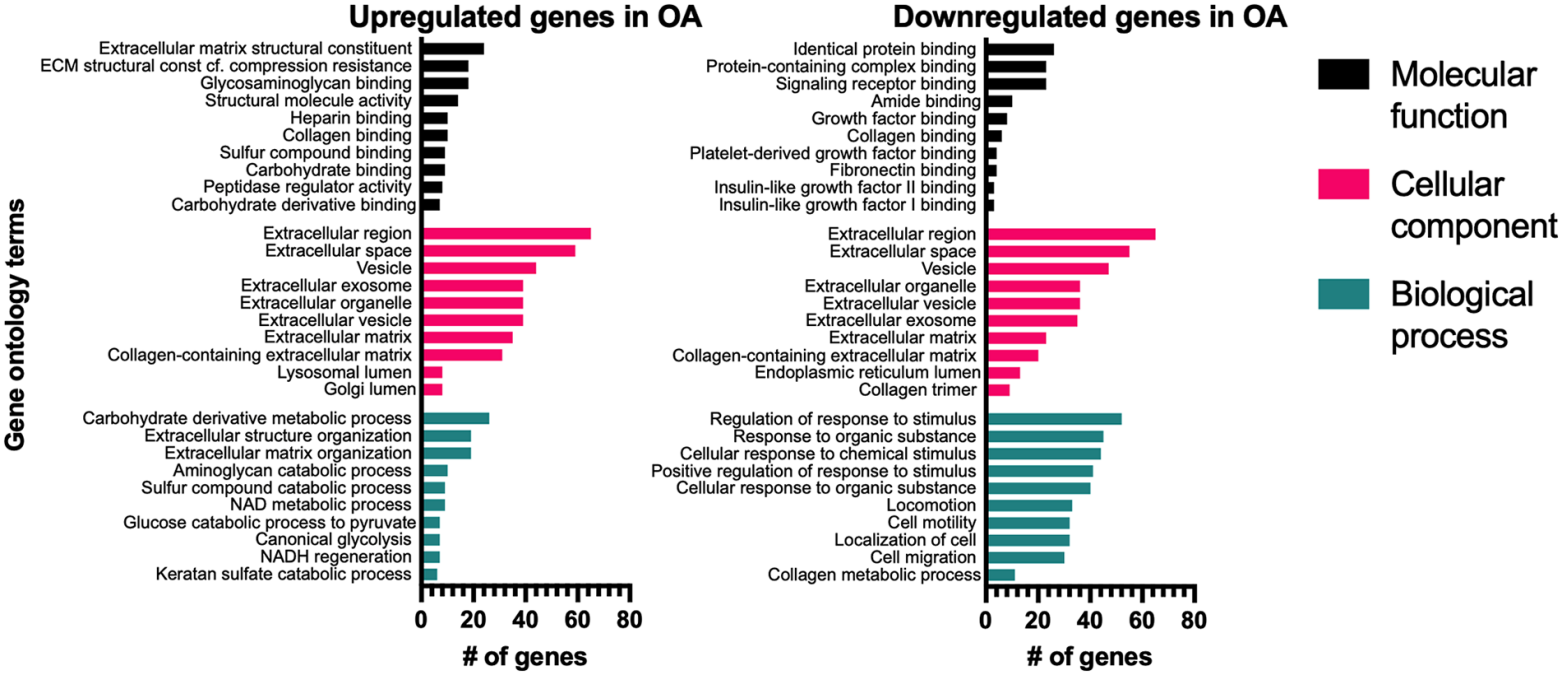
Bar graphs of enriched GO-terms. GO-term analysis of top 100 expressed genes significantly up- or downregulated in OA. The top 10 enriched terms shown for each domain (FDR < 0.01). Number of genes mapping to a particular term on x-axis. GO = Gene Ontology; OA = osteoarthritis; FDR = false discovery rate.

**Figure 4.**
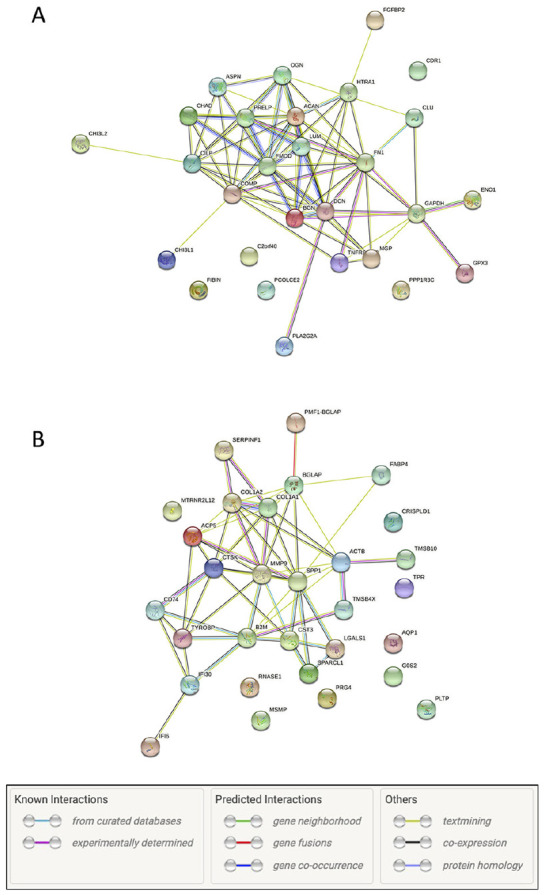
Graphical representation using STRING database (v.11.0) of protein-to-protein interactions of top 30 expressed and significantly (false discovery rate < 0.05) upregulated (panel A) or downregulated (panel B) genes corresponding to **Tables 3 and 4**.

When comparing the OA with the control group, there were 659 genes upregulated over 2-fold in OA and 1,369 genes downregulated (**Fig. 2**, Suppl. file 1). We sorted the identified genes for expression (**Tables 3 and 4**). Upregulated genes contained several known extracellular matrix molecules such as cartilage oligomeric matrix protein (COMP), cartilage intermediate layer protein (CILP), fibromodulin (FMOD), aggrecan (ACAN), lumican (LUM), and decorin (DCN). Downregulated genes also contained extracellular matrix constituents such as collagen I (COL1A1), lubricin (PRG4) but also MSMP, the same gene found downregulated in CL samples versus HC samples. Gene Ontology enrichment analysis performed using the 100 highest expressed genes (**Fig. 3**, Suppl. Fig. 1) confirmed that the GO-terms significantly enriched were involved with extracellular matrix regulation and organization as well as growth factor binding. To further visualize the potential networks between the identified regulated genes, we used the STRING database on the top 30 expressed genes upregulated and downregulated, respectively (**Fig. 4**). These plots confirm that the genes identified have known interactions with each other and only a few genes do not have any known links between them.

**Table 3.** Top 30 Expressed Genes with At Least a 2-Fold Upregulation between the Level Expressed in OA Compared with HC/CL Samples.

Gene	Fold Change	FDR	Normalized Count in OA
COMP	7.1	<0.001	14,294.4
CILP	8.1	<0.001	3,736.4
CLU	3.2	<0.001	3,357.2
FN1	5.2	<0.001	3,240.3
C2orf40	3.6	<0.001	2,378.0
MGP	3.5	<0.001	2,005.2
FMOD	4.1	<0.001	1,800.3
LUM	3.4	<0.001	1,359.4
BGN	2.4	<0.001	1,122.7
FGFBP2	2.6	0.003	1,047.6
RP5-940J5.9	2.3	<0.001	1,016.1
PRELP	5.8	<0.001	932.9
DCN	3.7	<0.001	930.1
CHAD	2.0	0.04	803.3
ACAN	4.1	<0.001	569.1
CHI3L1	4.2	<0.001	537.7
GAPDH	2.4	<0.001	453.2
PLA2G2A	4.8	<0.001	427.6
GPX3	2.9	<0.001	400.0
RP11-498C9.2	2.3	<0.001	356.9
CDR1	3.0	<0.001	353.0
PPP1R3C	4.6	<0.001	310.6
TNFRSF11B	13.0	<0.001	305.1
CHI3L2	8.3	<0.001	277.9
HTRA1	2.6	<0.001	252.6
OGN	4.7	<0.001	250.4
PCOLCE2	3.3	<0.001	234.2
ENO1	3.0	<0.001	232.5
ASPN	2.9	<0.001	211.1
FIBIN	2.8	0.001	206.7

HC/CL is treated as the control. Sorted for highest expression in OA samples.

OA = osteoarthritis; HC = healthy cartilage; CL = cartilage lesion; FDR = false discovery rate.

**Table 4.** Top 30 Expressed Genes with At Least a 2-Fold Downregulation between the Level Expressed in OA Compared with HC/CL Samples.

Gene	Fold Change	FDR	Normalized Count in Control
MSMP	–3.7	0.016	1,379.7
PRG4	–5.2	<0.001	742.7
COL1A2	–3.7	<0.001	659.3
ACTB	–2.1	<0.001	374.0
CTSK	–3.9	<0.001	365.6
COL1A1	–50.9	<0.001	344.7
SPP1	–3.7	0.024	260.0
TMSB10	–7.7	<0.001	258.9
CD74	–31.7	<0.001	150.5
TMSB4X	–10.9	<0.001	145.3
LGALS1	–4.4	<0.001	128.7
MTRNR2L12	–2.8	<0.001	128.0
CRISPLD1	–2.9	0.003	115.2
BGLAP	–85.7	<0.001	111.1
RNASE1	–82.1	<0.001	110.2
PMF1–BGLAP	–12.5	<0.001	107.4
SPARCL1	–147.6	<0.001	104.9
SERPINF1	–129.4	<0.001	80.3
FABP4	–31.0	<0.001	77.1
G0S2	–9.0	<0.001	76.2
TYROBP	–35.5	<0.001	67.5
CST3	–2.6	<0.001	66.3
MMP9	–56.9	<0.001	66.0
PLTP	–5.9	<0.001	65.6
IFI30	–26.2	<0.001	62.4
TPR	–4.2	<0.001	62.0
AQP1	–5.0	<0.001	61.8
IFI6	–2.1	<0.001	58.7
ACP5	–111.1	<0.001	58.4
B2M	–2.1	<0.001	53.5

HC/CL is treated as the control. Sorted for highest expression in control samples.

OA = osteoarthritis; HC = healthy cartilage; CL = cartilage lesion; FDR = false discovery rate.

#### Functional assays

**Figure 5.**
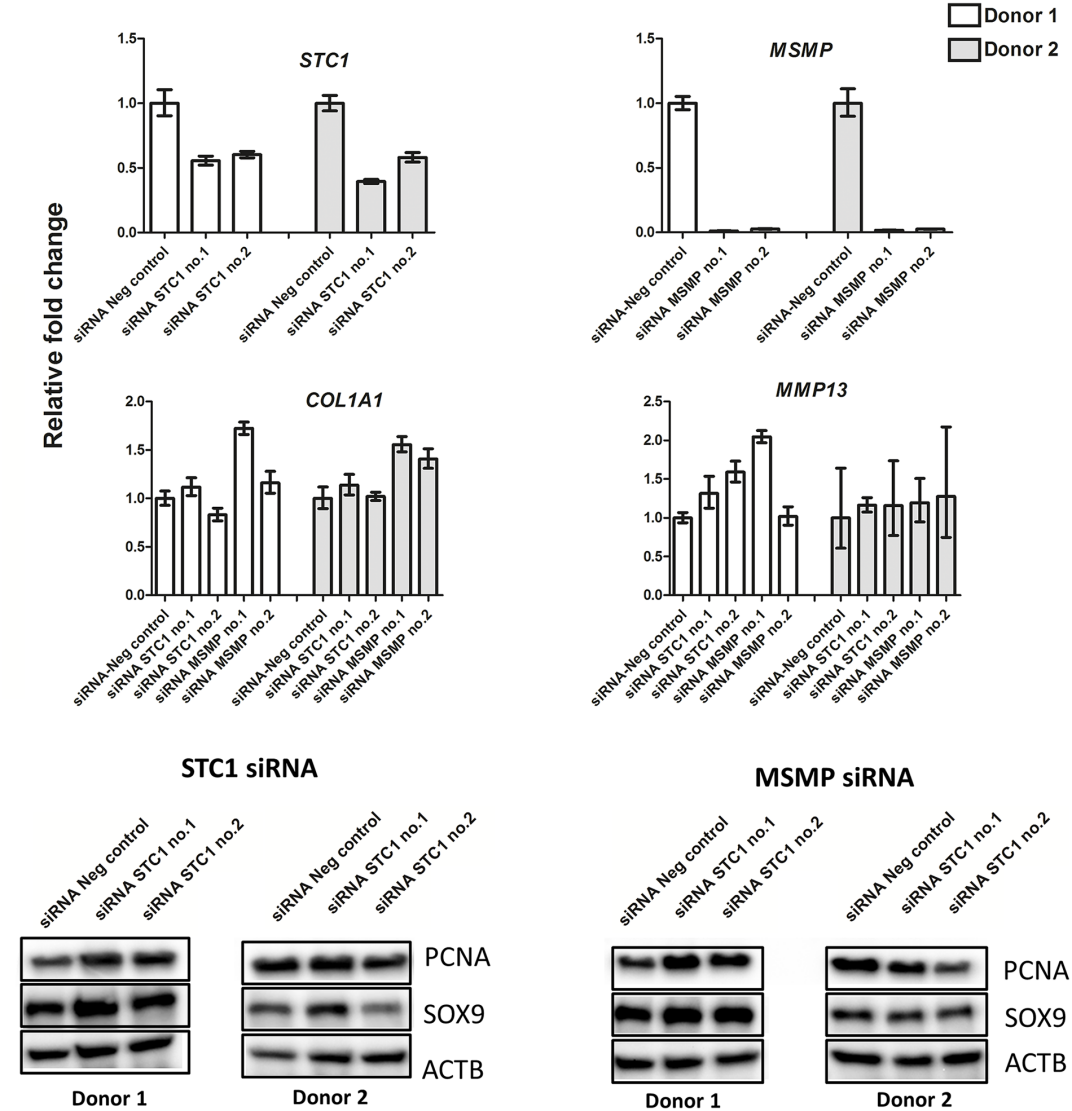
Knockdown of STC1 and MSMP by siRNA in articular chondrocytes. RT-qPCR in upper panels for STC1 and MSMP, in middle panels for MMP-13 and COL1A1 (GAPDH was used as control), and Western blots for PCNA and SOX9 with ACT as control in lower panels. PCNA = proliferating cell nuclear antigen.

To investigate the potential role in chondrogenesis of the findings from the sequencing data further, we chose two of the downregulated genes (one with high expression [MSMP] and one with low expression [STC1]) for functional assays. Using siRNA, we knocked down expression of both genes in cultured chondrocytes and examined the RNA expression of MMP-13 and COL1A1 without any apparent direct effect. We also performed protein expression assays of proliferating cell nuclear antigen (PCNA), a marker of cell proliferation and SRY-box transcription factor 9 (SOX9), a key chondrogenic transcription factor (**Fig. 5**). For STC1 we achieved approximately 50% knockdown in RNA expression and for MSMP more than 95% knockdown, however without a consistent effect on the chosen markers.

## Discussion

The treatment of both traumatic CLs and OA continues to present difficulties to both surgeons and patients. To our knowledge, this is the first study to compare gene expression of non-weightbearing cartilage between all 3 groups. The first important finding is that there were only very minor differences in gene expression between cartilage sampled from knees with a CL and without. In contrast, we found a markedly different expression of genes between the control group of HC and CL compared with OA.

There were very few differences between HC and CL gene expression, with only 6 genes that had at least a 2-fold change. We believe this is due to the CL samples being collected from a non-weightbearing area as opposed to directly from the lesion, as was done in a recently published study by Asik *et al.*^18^ that found significant differences between healthy and lesion cartilage within the same knee. This indicates that gene expression changes in CL knees are limited to the area surrounding the lesion; this is further supported by differences in gene expression between intact and damaged cartilage in OA knees.^1,35,36^ The low number of differences also supports that the localized CL is not part of a generalized degenerative process of the articular cartilage in the joint involved, as expected, and a successful cartilage repair could therefore be achieved. Interestingly, none of the 6 genes have fully known functions relating to cartilage. The MSMP gene had the greatest fold change and was downregulated 22-fold in CL. It has been characterized as prostate-specific and is abundantly expressed in benign and malignant prostate and breast tumors and to a lesser extent in ovarian and lung tumors.^37–40^ New research has identified the gene to be downregulated in equine OA chondrocytes^28^ and enriched in the chondrocyte module in human juvenile articular cartilage.^41^ It has also been proposed to be a potential target for the microRNA miR-140 which is linked to OA through the regulation of ADAMTS5.^26,42^ In a limited experiment with 2 donors, we tested the simple hypothesis that knockdown of MSMP in articular chondrocytes in culture could affect proliferation or chondrogenic markers (**Fig. 5**), with no clear effect on PCNA or SOX9.

Another downregulated gene in CL compared with HC was STC1, which was also downregulated, albeit from a much lower base level, 15-fold in the OA samples compared with the control group while its homolog STC2 was upregulated 7-fold. Gelse *et al.*^43^ found that expression of STC1 and STC2 was significantly higher in damaged tissue when comparing healthy articular cartilage with osteophyte/damaged cartilage. Lambert *et al.*^44^ identified STC1 as the most upregulated gene in inflamed synovial tissue compared with normal/reactive areas and hypothesized that STC1 could be a key mediator of synovium neovascularization in OA synovitis. Fernández-Torres *et al.*^45,46^ later found in 2 separate studies that the STC1 gene and gene-to-gene interaction between STC1 and COL11A1 were associated with knee OA susceptibility, while Wu *et al.*^47^ showed that upregulation of STC1 inhibited the development of OA by inhibiting survival and inflammation of fibroblast-like synovial cells and thereby may exert a protective role in OA. Similar to MSMP we did not find a clear effect of knockdown experiments using siRNA on PCNA or SOX9. However, as discussed here, both STC1 and MSMP may play a significant role in cartilage homeostasis or OA development and further studies are needed to identify their functions and potential as therapeutic targets.

Looking at OA in comparison with the HC/CL group, we found a large number of regulated genes. Both up- and downregulated genes mapped to cellular component GO-terms in the extracellular matrix domain. Downregulated genes mapped to biological processes of responses to stimulus, cell motility, and cell migration, while upregulated genes mapped to extracellular matrix structure and organization. The downregulated genes that were downregulated from high expression in HC/CL interestingly included MSMP, further underlining that MSMP may play a role in OA, but also several other genes such as PPARG, HYAL2, RUNX2, PRG4, and NOTCH1, which have all previously been shown to be related to OA processes (**Table 4** and Supplementary files).^48–52^ Matrix metallopeptidase 13 (MMP-13), which is a central node in the cartilage degradation network,^53–55^ was downregulated in the OA samples compared with the control group, with a fold change of almost 13. MMP-13 expression has been shown to vary throughout OA progression and is usually upregulated in the early stages and downregulated in the late stages.^11,35,55–57^ Furthermore, cartilage from knees with ACL-injury are also known to have upregulated expression of MMP-13 RNA,^58^ and as our HC samples were collected from patients undergoing ACL-reconstruction, this could have contributed to the significant downregulation in OA compared with the control group. Of the upregulated genes, a number of them have previously been associated with OA when upregulated, including TNFAIP6, LUM, NKX3.2, DCN, and PTGS2.^1,59–61^ Furthermore, FMOD, NGF, LOXL3, COMP, PTGES, and VEGFA were also upregulated in our OA samples and have all previously been established as OA genes.^62–67^

A factor we were not able to accurately track was the time from injury to operation, as not all patients had a concrete event or trauma, and some had experienced knee pain for years prior to seeking help. This unknown factor could also contribute to the lack of differences between HC and CL as Papathanasiou *et al.*^58^ demonstrated that macroscopically intact, non-weightbearing cartilage in knees undergoing ACL-reconstruction showed a correlation between increased levels of apoptotic, inflammatory, and catabolic factors with increased time from injury to operation.

Due to the inherent difficulties of working with cartilage which limit the amount of RNA that can be isolated per milligram of sample,^68^ as well as limitations in the amount of cartilage we could safely remove from HC and CL patients, we were not able to collect sufficient samples to validate our findings with PCR. Another possible bias with samples taken during surgery is contamination with blood. Expression levels of hemoglobin beta (HBB) were higher in OA samples than in HC and CL samples but still at very low level (Suppl. file 1). Another limitation we could not address was the significant difference in mean and range of ages and BMI between the groups. Due to anonymization of the samples prior to sending abroad for gene sequencing, we were not able to adjust for sex, age, or BMI in our analyses and thereby not able to explore which changes may be specific for age or BMI. Cartilage is known to become more susceptible to damage with increasing age and this could have affected our results as there was only a small overlap in age between the OA and control groups, and both age and BMI were significantly different.^12,69–71^ We acknowledge that other RNA-seq protocols such as the LIEA RNA-seq protocol^72^ could have been utilized reducing the risk of bias for smaller transcripts; however, at the time of the planning of the study, this was not available to us. A general caveat is also that with limited number of donors we may lack power to reliably detect genes with only small differences in expression.

One of the strengths of this study was the number of samples acquired, including samples of HC from live donors. Samples of HC from living, non-amputation, or non-OA donors are especially difficult to acquire due to the ethical considerations of removing a sample from HC. We chose to harvest samples using the same method and location as for ACI. ACI is a well-established method for treating CLs and has been in use for over 20 years with improvement in quality of life reported in short-term and intermediate-term outcome studies.^73^ Previous studies for donor site morbidity have identified no significant difference in Lysholm scores up to 5 years post-harvest in asymptomatic knees^74^ and found no problem areas on MRIs in the middle or long term.^75^ However, our choice to harvest non-weightbearing cartilage for ethical reasons may have affected the results as mechanical forces are a well-established factor in OA development.^12,76^ The gene expression profile of the HC will be made available open access.

## Supplemental Material

sj-tif-1-car-10.1177_19476035211057245 – Supplemental material for Low-Input RNA-Sequencing in Patients with Cartilage Lesions, Osteoarthritis, and Healthy Cartilage Click here for additional data file. Supplemental material, sj-tif-1-car-10.1177_19476035211057245 for Low-Input RNA-Sequencing in Patients with Cartilage Lesions, Osteoarthritis, and Healthy Cartilage by Katherine Wang, Q.Y. Esbensen, T.A. Karlsen, C.N. Eftang, C. Owesen, A. Aroen and R.B. Jakobsen in CARTILAGE

sj-xls-1-car-10.1177_19476035211057245 – Supplemental material for Low-Input RNA-Sequencing in Patients with Cartilage Lesions, Osteoarthritis, and Healthy Cartilage Click here for additional data file. Supplemental material, sj-xls-1-car-10.1177_19476035211057245 for Low-Input RNA-Sequencing in Patients with Cartilage Lesions, Osteoarthritis, and Healthy Cartilage by Katherine Wang, Q.Y. Esbensen, T.A. Karlsen, C.N. Eftang, C. Owesen, A. Aroen and R.B. Jakobsen in CARTILAGE
