# Supplementary figures and images for: Low-Input RNA-Sequencing in Patients with Cartilage Lesions, Osteoarthritis, and Healthy Cartilage
Source: Cartilage. 2021 Nov 15;13(1 Suppl):550S–562S. doi: 10.1177/19476035211057245 (PMC8808811; doi:10.1177/19476035211057245)

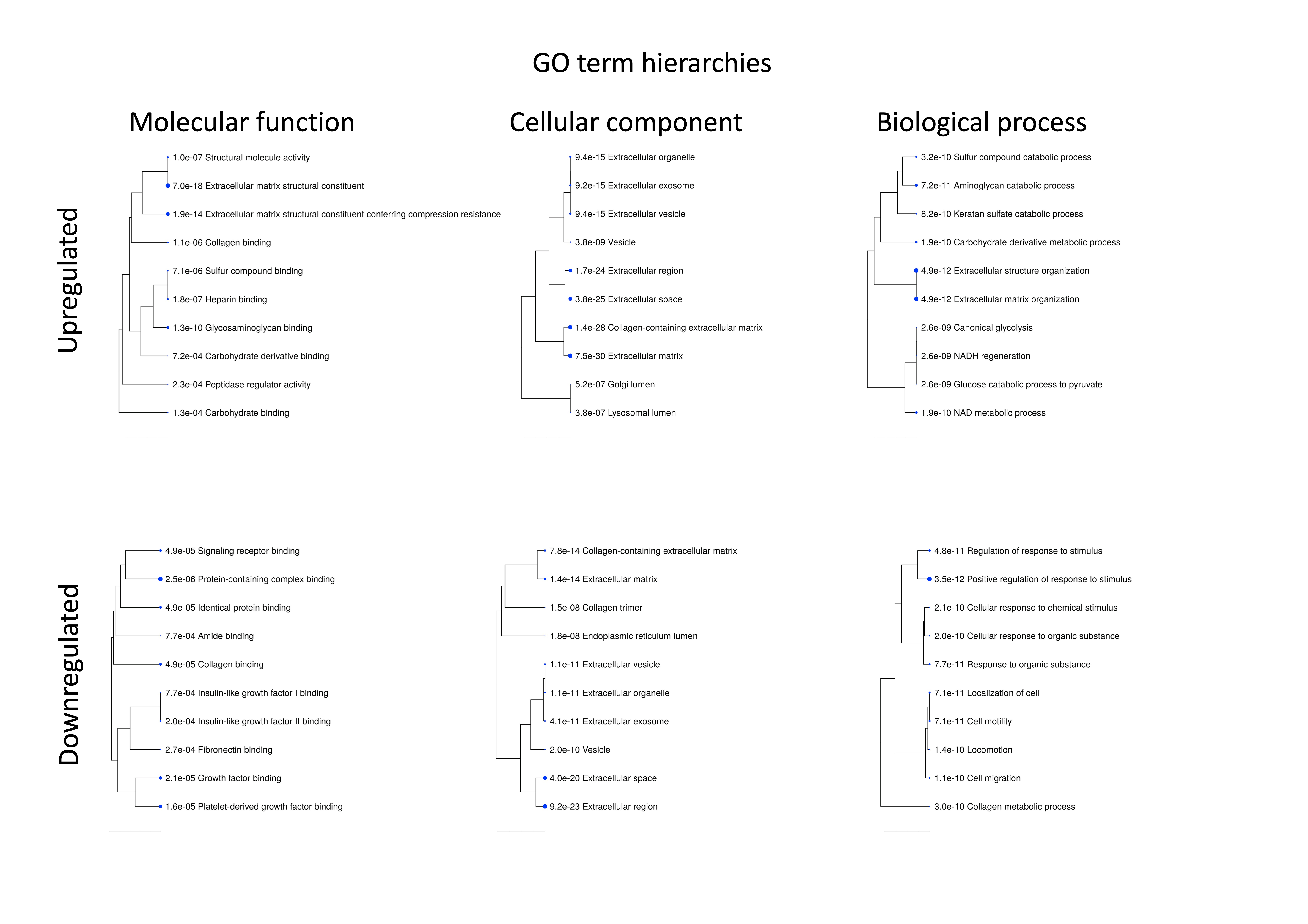

Supplement: sj-tif-1-car-10.1177_19476035211057245 – Supplemental material for Low-Input RNA-Sequencing in Patients with Cartilage Lesions, Osteoarthritis, and Healthy Cartilage [file sj-tif-1-car-10.1177_19476035211057245.tif]
